# Enhanced photo-reactivity of polyanthracene in the VIS region

**DOI:** 10.1371/journal.pone.0271280

**Published:** 2022-07-08

**Authors:** Dwight Angelo Bruzon, Anna Pamela De Jesus, Chris Dion Bautista, Imee Su Martinez, Monissa C. Paderes, Giovanni A. Tapang

**Affiliations:** 1 Materials Science and Engineering Program, College of Science, University of the Philippines Diliman, Quezon City, Philippines; 2 Institute of Mathematical Sciences and Physics, College of Arts and Sciences, University of the Philippines Los Banos, Laguna, Philippines; 3 National Institute of Physics, College of Science, University of the Philippines Diliman, Quezon City, Philippines; 4 Institute of Chemistry, College of Science, University of the Philippines Diliman, Quezon City, Philippines; The University of Tokyo, JAPAN

## Abstract

The wavelength-dependent photo-reactivity of polyanthracene was explored upon UV-C and VIS light irradiation. The material was prepared via one-pot chemical oxidation route using FeCl_3_ as oxidizing agent. A decrease in surface hydrophobicity of a polyanthracene-coated poly(methylmethacrylate) substrate from 109.11° to 60.82° was observed upon UV-C exposure for 48 hrs which was attributed to increase in oxygen content at the surface, as validated by energy dispersive X-ray spectroscopy. Upon exposure to ultraviolet-visible LEDs, photo-dimerization of polyanthracene in solution occurred and was monitored using UV-VIS spectroscopy. The photo-dimer product formation decreased from 381 nm to 468 nm and was found to be higher for the polyanthracene material compared to the monomer anthracene. At 381 nm, photo-dimerization of the material was found to be approx. 4x more efficient than the non-substituted monomer counterpart. Results obtained show that photo-dimerization of polyanthracene will proceed upon exposure with visible light LEDs with reduction in efficiency at longer wavelengths. To compensate, irradiation power of the light source and irradiation time were increased.

## Introduction

Intelligent molecular systems have properties or functions that can be switched using specific interventions to perform desired tasks or work. These interventions can be physical in nature, such as variation in electric or magnetic field, application of compressive or tensile mechanical force, and exposure to light at different wavelengths or energy [1–3]. Chemical interventions can also provide molecular control over materials such as variations in pH or applied potential for oxidation-reduction coupled reactions. For instance, by adjusting the pH or temperature of an aqueous solution containing hydrogels, the degree of swelling can be controlled to favor either the stretched, hydrated polymer chain or the collapsed, hydrophobic structure [4]. These molecular systems have been explored for applications such as controlled drug release agents, tissue engineering, bio-sensing, smart coatings, artificial muscles, self-healing materials and more [5–8].

Stimuli-responsive moieties enable controlled response of these materials from applied external stresses [9]. The use of light, in particular, as external stimulus is driven by various motivations. Its sustainability and biocompatibility made light a desired energy source over thermal switching. It is also non-invasive and non-destructive and allows remote localization and delivery of energy to a system. This spatio-temporal control allows more flexibility in terms of switching material properties over thermal, magnetic, and electric stimuli. Photo-driven chemical reactions have been explored in the development of smart functional materials by absorbing energy which can induce physical or chemical changes on the material [10]. These changes which occur via photo-activated mechanism allows rapid, efficient, and economical means of controlling material properties, which can be tuned by varying parameters of the input light such as exposure wavelength, irradiation time, and power.

The light-mediated chemistry of anthracene moieties is one of the widely explored and utilized photo-activated switching in various materials [11, 12]. Concerted rearrangement of molecular bonds between two anthracene molecules upon exposure to λ_1_, typically at wavelengths >300 nm, enables formation of anthracene photo-dimers which occur via photo-induced [4+4] cycloaddition mechanism. Photo-cleavage, or the breaking of a photo-dimer, may be induced either upon exposure to λ_2_, typically at wavelengths <300 nm, or heat (Δ) [13, 14]. Cyclo-reversion may also be induced by mechanical means, extending its applications as mechanopores [15]. It can undergo dimer formation and scission for a repeated number of cycles without undesired side reactions such as isomerization and hydrolysis [16].

Anthracene dimerization has been utilized in a wide variety of applications. As a photo-active substituent, anthracene has been used as coupling or cross-linking agent, and in processes such as polymerization, chain extension, surface grafting, surface patterning, self-healing, and polymer cyclization [17–20]. Self-healing materials based on ion gels, hydrogels, and polymers were developed using anthracene moieties tethered to the polymer backbone or dendritic branches of the material of choice [7, 21, 22]. Bio-orthogonal hydrogels were modified with anthracene groups as catalyst-free initiators for cross-linking, forming bulk hydrogels and microgels and improving cell bioconjugation [23]. Anthracene-modified materials have also been used to create reversible functionalized surfaces driven by visible light for writing and ultraviolet light for erasing [24]. Shifting the dimerization wavelength of anthracene to the visible region has been made possible by functionalizing anthracene with triazole, an electron-donating substituent, at the C9 position of the polyaromatic ring [23, 24]. This offers biocompatibility for directing or guiding cell adhesion and growth. To date, only by functionalizing anthracene-containing molecular systems with triazole has the red-shift in the photo-dimerization and photo-scission wavelengths been observed experimentally. Reactivity plots or spectrum of these materials are needed to determine with accuracy the wavelengths of interest [25].

To this end, we investigate the photo-reactivity of the material polyanthracene, a linear polymer which consists of an anthracene backbone linked at C9 and C10, in the UV-C (254 nm) and ultraviolet-visible (381–468 nm) regions. These regions are of high interest because red-shift in the dimerization wavelength in functionalized or substituted anthracene-based materials has only been pushed in the high-frequency visible region, and majority of studies involving photo-cycling these materials utilize UV-C as the stimulus for inducing dimer photo-scission. The polymer was prepared via chemical oxidation route, using anhydrous FeCl_3_ in nitrobenzene as oxidizing solution. The study intends to provide a reactivity spectrum for the material by characterizing the changes arising from UV-C and visible light exposure of polyanthracene.

## Materials and methods

The monomer anthracene, C_14_H_10_, was purchased from Aldrich (141062, ReagentPlus, ≥99%). The oxidizing agent anhydrous ferric chloride, FeCl_3_, was obtained from Techno Pharmachem (≥96%), and was stored in a reduced-humidity dry box until use since the reagent is highly hygroscopic. The solvent nitrobenzene, C_6_H_5_NO_2_, was purchased from Sigma Aldrich (2520379, ACS reagent, ≥99.9%) while n-hexane and absolute ethanol were purchased from Merck Germany. All reagents were used without further purification.

**Fig 1:**
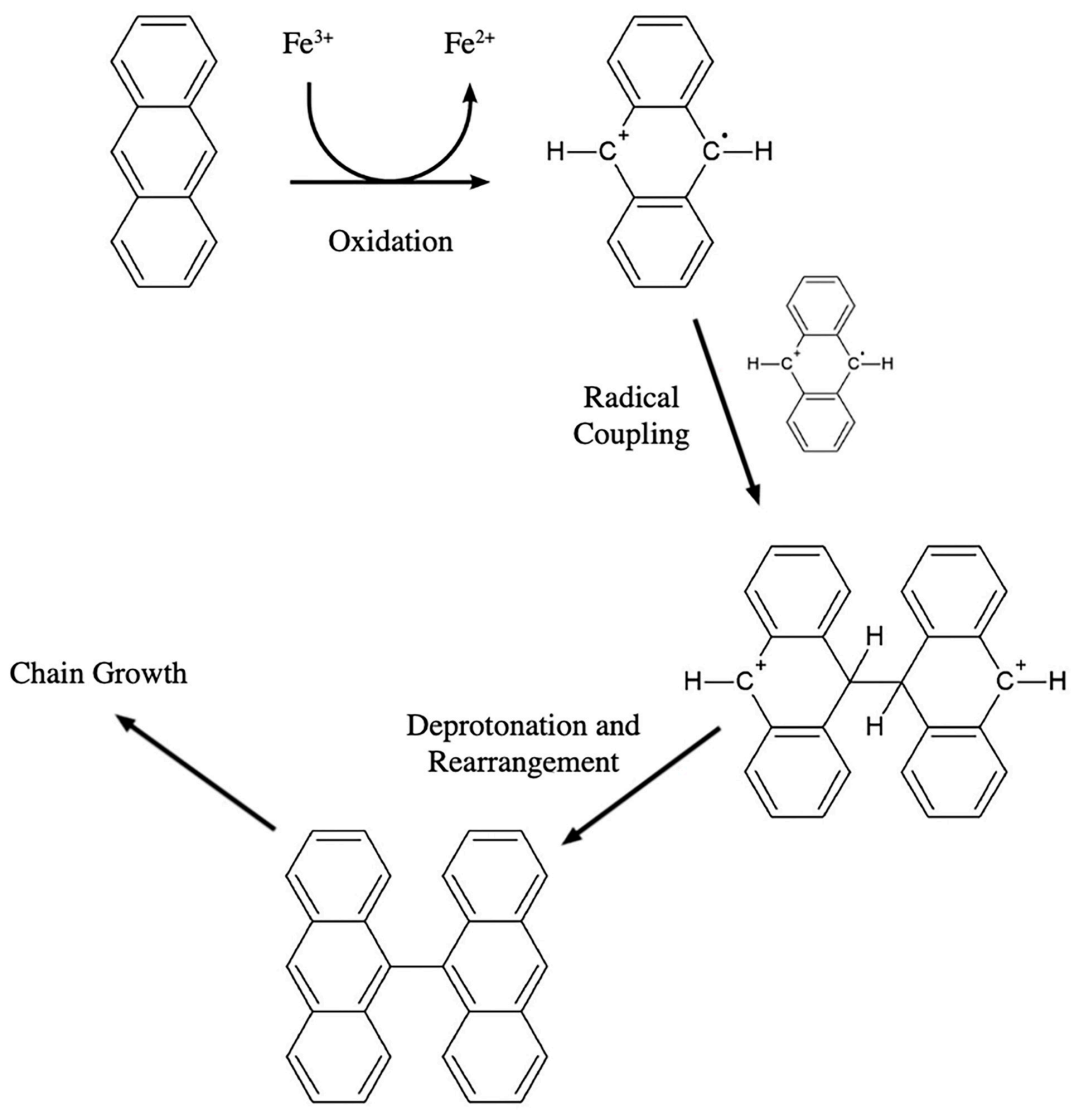
Formation of polyanthracene via chemical oxidative route.

Synthetic route was via one-pot chemical oxidation of anthracene monomers to produce a linear polyanthracene (p-An) chain as shown in Fig 1 [26–28]. A 0.15 M solution of anthracene in n-hexane was prepared and stirred at room temperature. Afterwards, anhydrous FeCl_3_ (0.33 M) dissolved in nitrobenzene was added to the solution, and the resulting mixture was heated to 60°C for 24 hours. A 2.2 mole equivalent of the oxidant solution was used since a lower ratio was reported to decrease the yield of the reaction [26]. Oxidative polymerization proceeds via coupling of the generated monomer radical cations formed by one-electron oxidation of anthracene [29, 30]. Repeat oxidation of the dimers formed after radical-radical coupling allows chain growth of the polymer backbone. The reaction was conducted in a non-aqueous environment because water can react with the radical cations and induce premature chain termination [31]. The reaction was terminated with ethanol. The solution was filtered with a Whatman filter paper grade 5 (Cat. No. 1005–110). The solid residue obtained was repeatedly collected, washed with distilled water, and filtered, until the filtrate tested negative for the presence of Fe^3+^ and Fe^2+^ ions, and chloride (Cl^-^) content was reduced to <10 ppm. Chloride content was measured using a calibrated ion-selective electrode. After washing with water, the solid residue was washed with ethanol three times, filtered, collected, and heated under low heat to dry the solid powder. Product obtained was a fine, brown powder with yield of >70%.

For material characterization, electron microscopy was conducted to determine structural morphology of the prepared polyanthracene using an FEI Helios Nanolab 600i Scanning Electron Microscope with 15.0 kV accelerating voltage and 0.69 nA beam current. An elemental analysis was done using energy dispersive X-ray spectroscopy (EDS) to provide detailed chemical composition of the polymer material. EDS was done using a Hitachi S-3400N Scanning Electron Microscope. The sample was sputtered with Au-Pd thin film coating using a Hitachi E-1010 Ion Sputter for 60 seconds prior analysis. Infrared spectrum was obtained using a Shimadzu IRPrestige-21 Fourier Transform Infrared Spectrophotometer with a PIKE MIRacle Attenuated Total Reflectance Accessory. UV-VIS absorbance spectrum was obtained using a double-beam UV-1700 PharmaSpec UV-VIS spectrophotometer. The molecular weight of the polymer was estimated via viscometry using a Brookfield DV2T Viscometer.

Static sessile drop contact angle measurements were done on polyanthracene coated on polymethylmethacrylate (PMMA). A 0.1% (w/v) polyanthracene dissolved in chloroform was prepared and coated on 10mm Ø PMMA thin sheets using a custom-build spin-coater. Contact angle of distilled water at the surface were obtained at 12-hr intervals for 48 hours upon UV-C exposure. Images obtained were analyzed using ImageJ.

To determine extent of photo-dimerization as a function of irradiation wavelength of the input light, a 0.003% (w/v) polyanthracene solution in ethanol was prepared in scintillation vials. The vials were placed in the sample chamber in the exposure set-up shown in S1 Fig. The light source for the exposure set-up was a 2x2 LED array arranged in a parallel circuit. Each vial was exposed to light-emitting diodes with peak wavelengths at 381, 392, 404, 462, and 468 nm for 60 mins. The emission spectra of each LED light source used was characterized using a Thorlabs CCS200 Compact Spectrometer and shown in S2 Fig. The voltage output of the DC power supply was adjusted so that the LED light sources provide a fixed power rating of 20 mW, measured by a digital power meter (PM100D, Thorlabs) with a silicon photodiode sensor (S120VC, Thorlabs) calibrated at 200–1100 nm. The extent of photo-dimerization of polyanthracene in each solution upon light exposure was quantified by measuring its absorbance using UV-VIS spectroscopy and validated via dynamic viscosity experiments.

## Results and discussion

**Fig 2:**
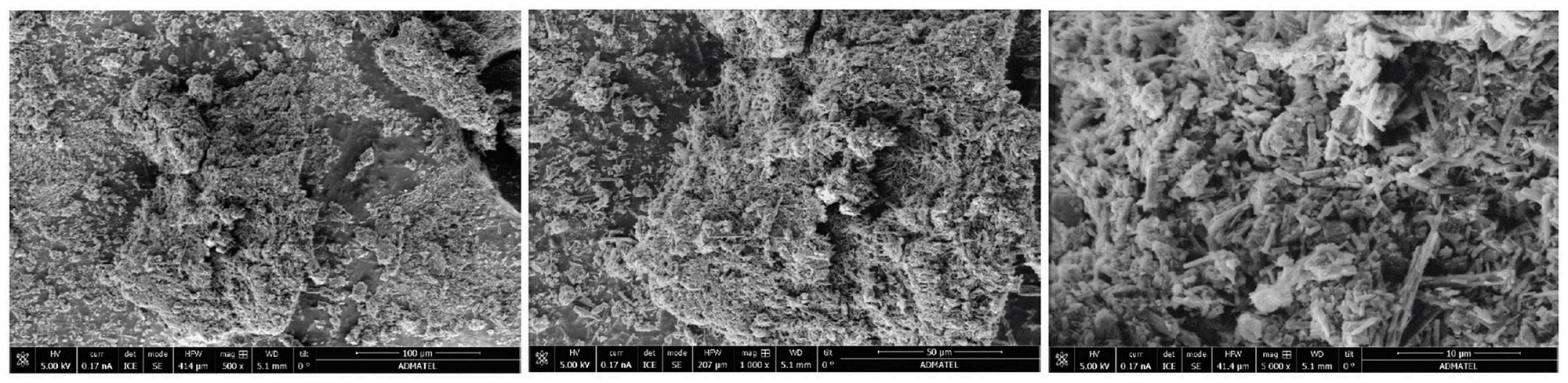
Scanning electron microscope images of prepared polyanthracene. Magnification of 500x (left), 1000x (middle), and 5000x (bottom).

The prepared polyanthracene was imaged using a scanning electron microscope. Fig 2 shows the SEM images of the material obtained under different magnifications. Images show densely packed polyanthracene with rough structural features. These observed geometric features can influence the surface wettability of polyanthracene. The EDS spectrum confirmed the presence of the elements Carbon (C), Oxygen (O), Chlorine (Cl), Iron (Fe), Palladium (Pd), and Gold (Au). Table 1 shows the chemical composition obtained in weight and atomic percent for the material.

**Table 1:** Elemental composition from EDS spectrum of prepared polyanthracene.

Element	Weight %	Atomic %
**Carbon / C (K)**	82.12	90.71
**Oxygen / O (K)**	9.72	8.06
**Chlorine / Cl (K)**	0.38	0.14
**Iron / Fe (K)**	2.56	0.61
**Palladium / Pd (L)**	2.11	0.26
**Gold / Au (M)**	3.12	0.21

From the table, the sample is mostly composed of C at 90.71 atomic %, which is expected since the polymer backbone is a linear chain of fused benzene rings. Presence of Au (0.21 atomic %) and Pd (0.26 atomic %) was due to the coating material used in the sputtering process. Iron (Fe) and Chlorine (Cl), present at atomic compositions 0.61% and 0.14%, respectively, were due to the oxidant solution prepared to facilitate the polymerization process. This may be due to incorporation of dopant ions in the polymer matrix during chain propagation. Other materials prepared via the same oxidative coupling polymerization, such as polypyrrole, using FeCl_3_ as oxidizing agent report incorporation of dopant ions such as FeCl_4_^-^ in the polymer matrix [32]. Tanemura and co-workers synthesized polyanthracene via chemical oxidation route using FeCl_3_ and reported a nominal sample composition of 0.60 Cl per anthracene unit [26]. Since nitrogen peaks were not observed in the EDS spectrum, the purification protocol employed in the study ensured complete removal of the solvent nitrobenzene. The oxygen content, at 8.06 atomic %, therefore may be attributed to water adsorbed by the material or oxidation products along the polymer chain.

**Fig 3:**
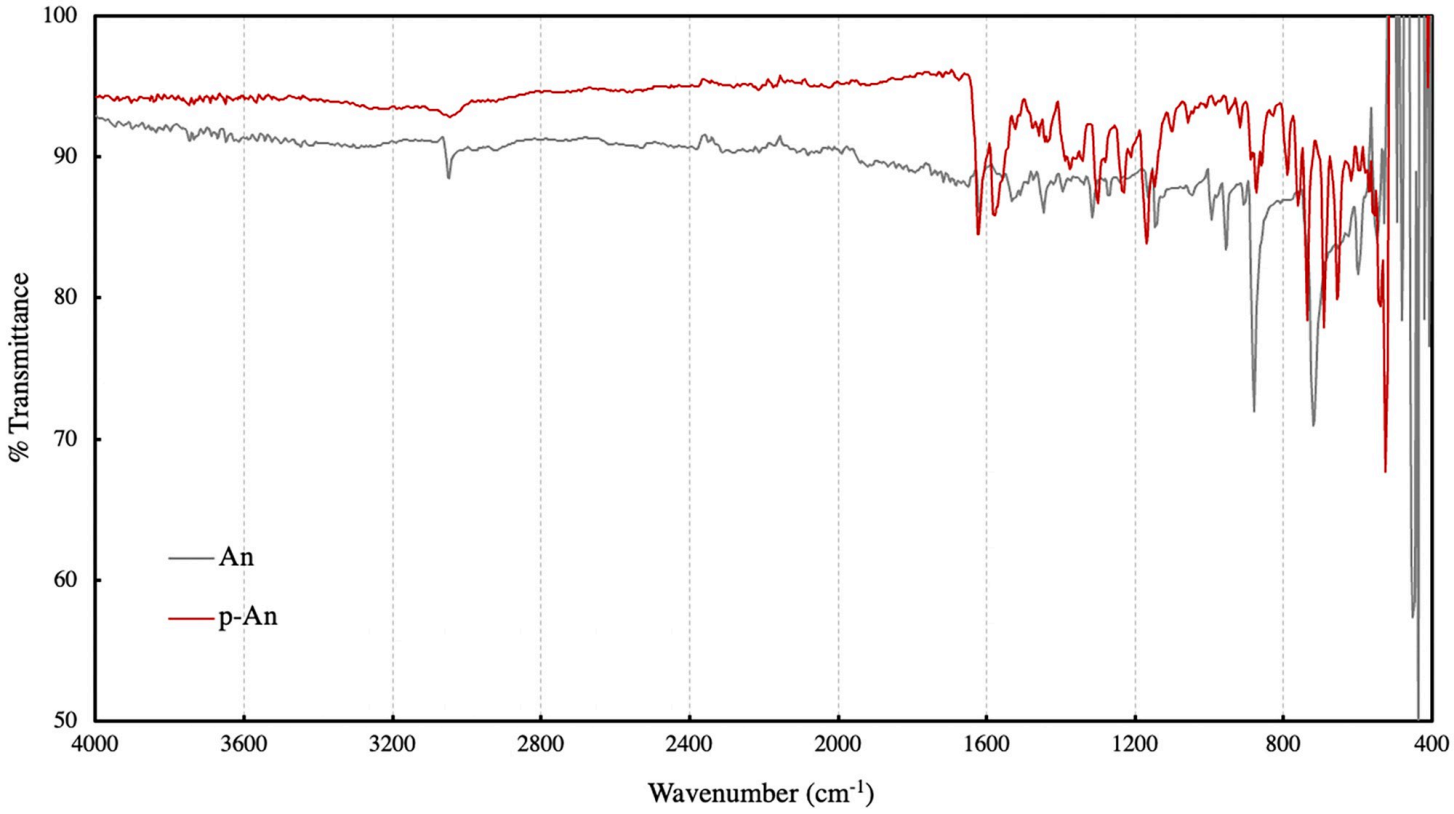
Infrared spectra of anthracene and polyanthracene.

The infrared spectra of the prepared polyanthracene and the monomer anthracene are presented in Fig 3. Aromatic C-H stretch for both anthracene and polyanthracene were observed at 3047 cm^-1^. Vibrational C-C stretches from the conjugated carbon backbone were observed at 1620 and 1578 cm^-1^. Also, a prominent C-H stretch at 883 cm^-1^ in the fingerprint region assigned to the vibrational stretch at the C9 and C10 positions decreased in the spectrum of the polymer, suggesting that coupling of the monomers occurred at these positions [29].

**Fig 4:**
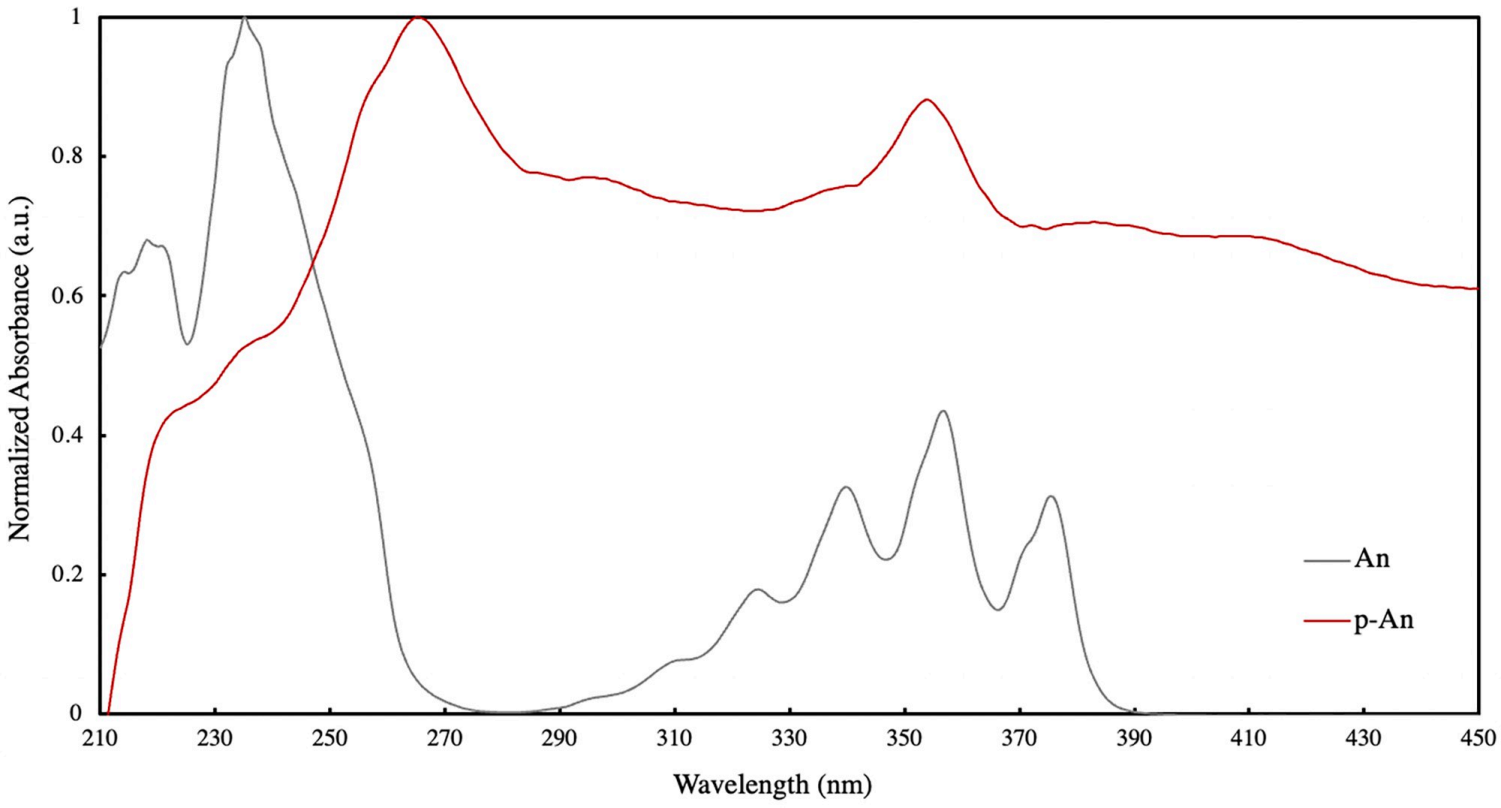
UV-VIS absorption spectrum of 0.003% w/v anthracene and polyanthracene in ethanol.

The UV-visible absorption spectrum of polyanthracene in ethanol shown in Fig 4 and is comparable with that reported in literature [27]. It exhibits broader and red-shifted absorption peaks at wavelengths 262 and 356 nm, in contrast to the well-structured spectrum of anthracene with absorption peaks within the regions 230–270 nm and 330–390 nm due to corresponding π-π* transitions. Absorption peak at 265 nm can be associated to an ortho-substituted benzene, such as o-xylene with absorption peaks at 212 and 262 nm and may thus present evidence of photo-dimerization in solution [33]. Since the material is polymeric in nature composed of multiple photo-active sites along the polymer backbone, the resulting dimerization upon light exposure may induce material self-aggregation, from an extended polymer conformation to a contracted or coiled conformation, or aggregation between two adjacent polymer chains.

The molecular weight of the synthesized material was estimated using viscometry. From the viscosity data of solutions of the reference standards polystyrene dissolved in ethyl acetate, the intrinsic viscosity was derived by extrapolating the plot of reduced viscosity in the limit of zero concentration and plotted against the molecular weight of the standards used, following Mark-Houwink equation. The calibration curve obtained is presented in S3 Fig. From the obtained curve, the viscosity-average molecular weight of the synthesized polyanthracene was found to be M_w_ 2,829 with degree of polymerization (DP) of 15.87 or 16. The results were close to reported literature value of M_w_ 2,321 with DP of 13 units which was obtained via Gel-Permeation Chromatography, albeit difference in reported characterization techniques [27].

**Fig 5:**
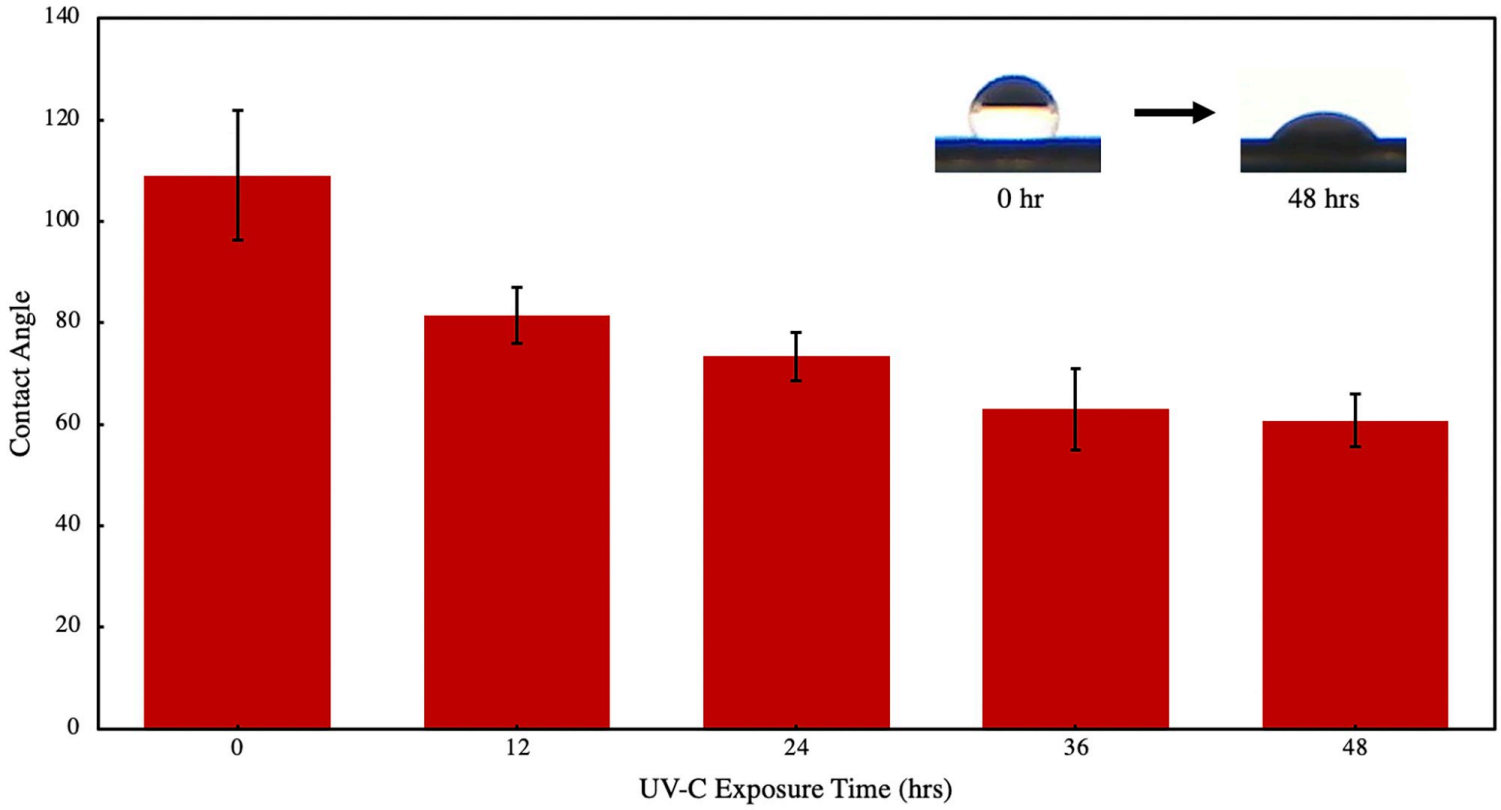
Contact angle of water on polyanthracene-coated PMMA upon exposure to UV-C over time. Inset shows actual change in surface wettability after 48 hrs of exposure.

To characterize effect of UV-C exposure on the material, static sessile drop contact angle experiment was conducted using distilled water on a polyanthracene-coated polymethylmethacrylate (PMMA) surface. Fig 5 shows the variation in contact angle of water upon exposure of the surface to UV-C light (peak λ @ 254 nm) over 48 hrs. The rough structural features of polyanthracene from Fig 2 may imply low hydrophobicity due to physical or geometric factors. However, it was observed that the polyanthracene-coated PMMA surface exhibits high hydrophobicity with a contact angle of 109.11° (±12.7), suggesting that chemical forces have a stronger influence on the observed wettability than physical or geometric. Contact angle of water was observed to decrease with longer UV-C exposure time of the material, dropping to a value of 60.82° (±5.15) after 48 hrs. This observed change in surface wettability may be attributed to an increase in the oxygen content of the material at the surface.

Exposure to UV-C can catalyze formation of singlet oxygen which can react via [4+2] Diels-Alder cyclo-addition to anthracene moieties, forming anthracene endoperoxides [14]. You and co-workers reported a methylene-bridged anthracene polymer wherein the hydrophobicity of the material decreased upon exposure to ultraviolet light due to formation of photo-oxidation products competing with the photo-induced dimerization of anthracene moieties [34]. Surface wettability of the material changed by 30° upon 10 mins of UV exposure. Observed faster kinetics of formation of photo-oxidation products compared to polyanthracene may be attributed to less steric strain-inducing substituents present at C9 and C10 of the reactive anthracene group [35]. It is also worth noting that electron-donating and electron-withdrawing character, and position of the substituents can both affect a material’s reactivity and its kinetics [36].

**Fig 6:**
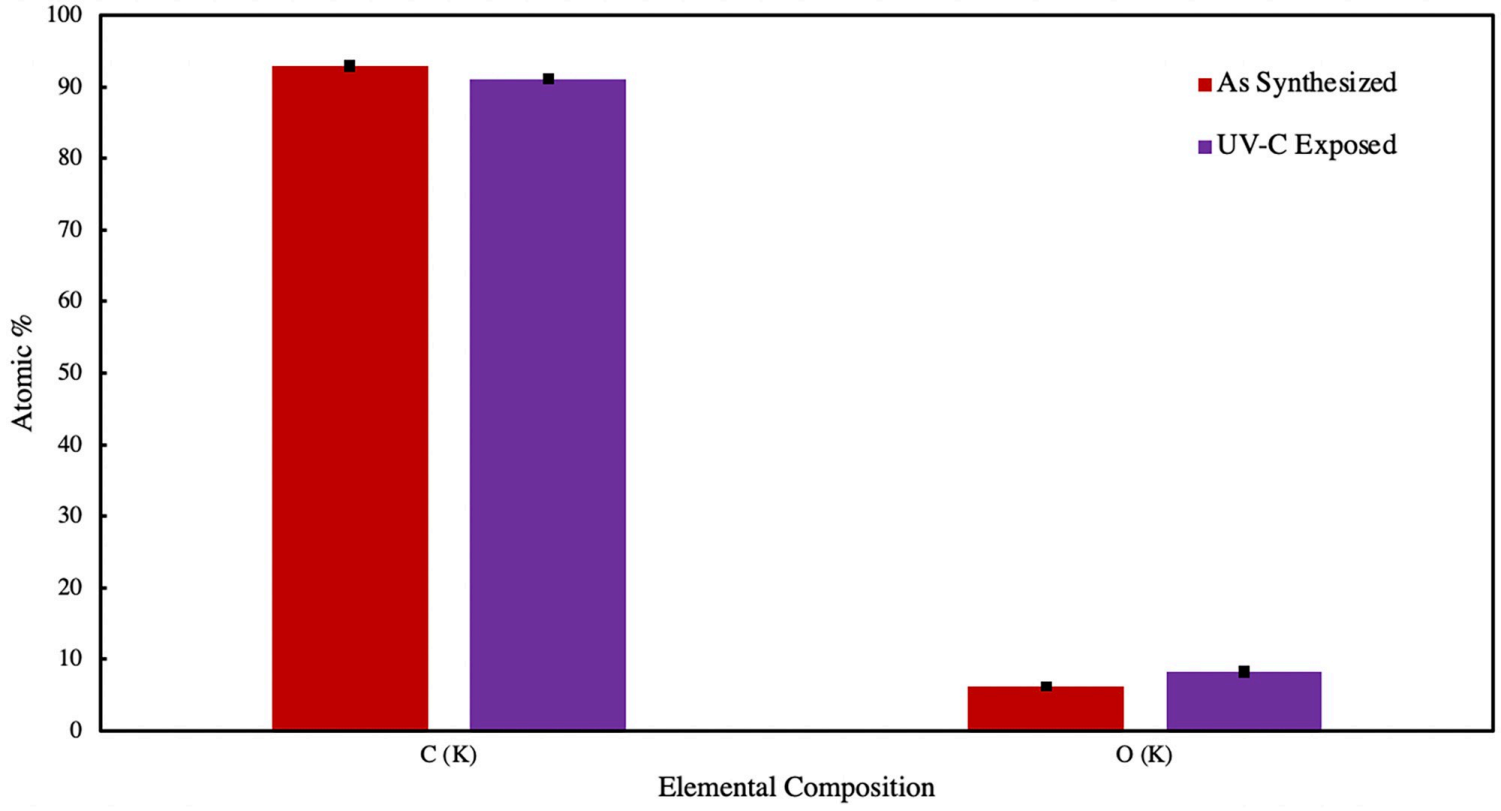
Elemental composition of polyanthracene before and after UV-C exposure. Before exposure (red), after exposure to UV-C for 24 hrs (purple).

Fig 6 shows the elemental composition obtained via energy dispersive x-ray spectroscopy for as-synthesized and UVC-exposed polyanthracene material. Chlorine (Cl) and Iron (Fe) were present at atomic percentages <0.5%. A notable increase of 2% in oxygen content, from 6.26 to 8.23 atomic %, was observed upon UV-C exposure for 24 hours, validating the surface wettability studies performed. A [4+2] Diels-Alder cyclo-addition reaction between polyanthracene and atmospheric oxygen proceeds upon exposure of the material to UV-C [14]. Photo-dissociation of anthracene endoperoxide can further proceed to form a more stable product anthraquinone. Some polymers, such as polystyrene, also report significant photo-oxidative degradation upon exposure to far-UV radiation, which results in breakage of polymer chains, production of radicals, reduction in molecular weight, and deterioration of mechanical properties [37]. In the interest of leveraging the reversible photochemistry of anthracene as a switchable moiety, formation of anthraquinone is an undesirable side reaction due to its irreversibility. Anthraquinone, however, has been found useful for other applications such as oxygen-reduction redox mediators for energy electrochemistry and oxidative photo-catalysis [38–41]. Similar studies exploiting the reversible lock-and-release chemistry of anthracene-functionalized materials utilize low energy UV light as stimulus for inducing photo-dimerization and heat as stimulus for inducing scission to avoid inactivating the central ring of the anthracene group [13, 19, 42, 43]. If photo-scission via high energy UV light is preferred, it is preferentially done under high vacuum or in the presence of an inert gas to limit exposure of the material to atmospheric oxygen [21, 44, 45].

**Fig 7:**
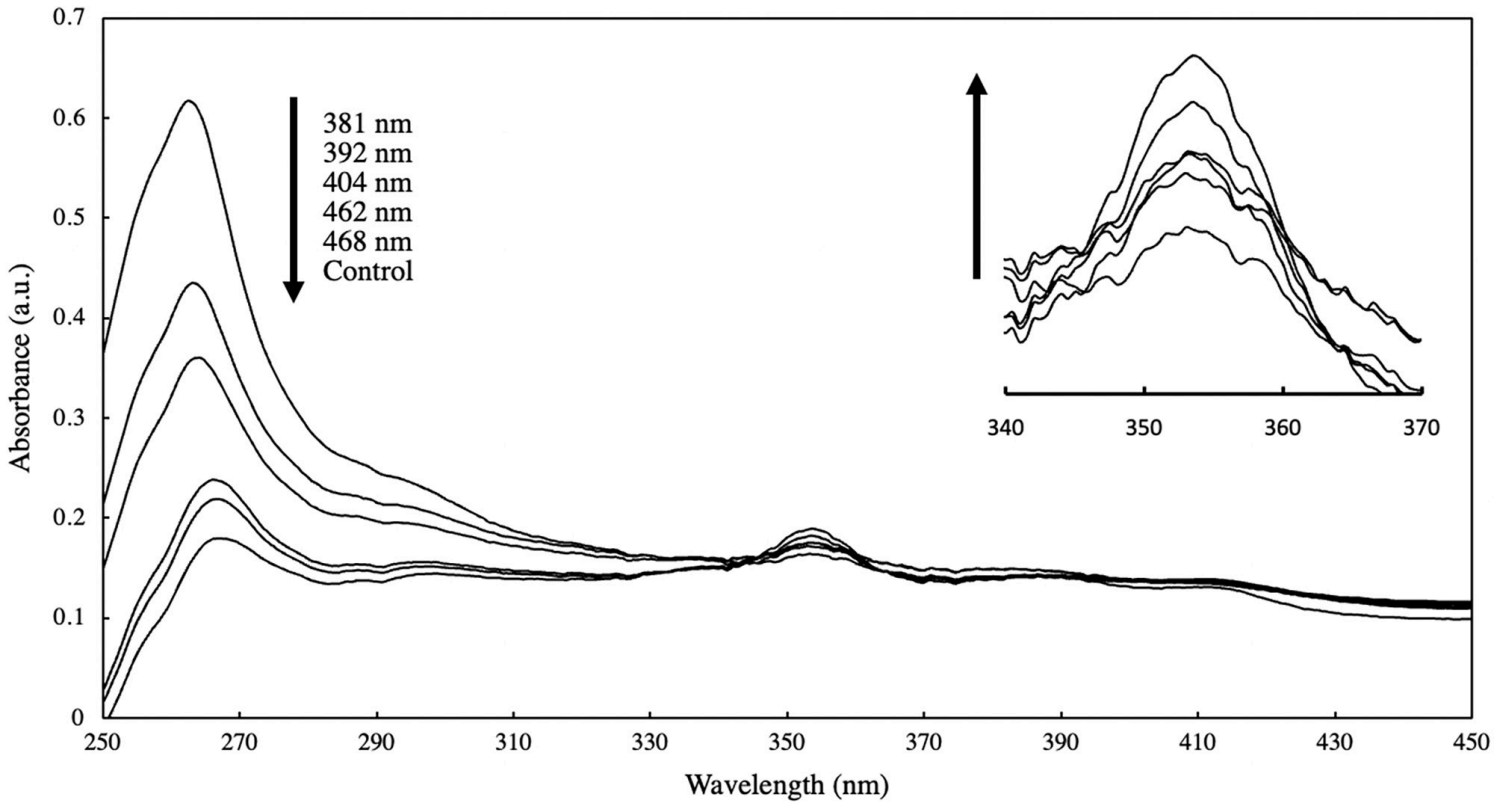
Absorbance spectra of 0.003% (w/v) polyanthracene in ethanol solutions exposed to different LED lights.

The material’s reactivity was characterized upon exposure to 381–468 nm light as stimulus. The wavelength-dependent photo-induced reactivity of polyanthracene was evaluated by monitoring the UV-VIS absorbance spectrum of the material. It is worth noting that the absorbance spectrum and its inherent photo-reactivity may not necessarily be coherent [25]. However, it can be utilized to monitor progress of photo-induced dimerization and scission of anthracene moieties, along with other techniques such as ^1^H-NMR and Infrared Spectroscopy [33, 43]. Monitoring dimerization and scission reactions among light-responsive moieties such as anthracene is of high interest to provide quantitative information on reaction kinetics, reactivity spectrum, and effect of substituents, especially for functionalized materials. Solutions of polyanthracene in ethanol were exposed to the different wavelengths of input light to induce cross-linking by photo-dimerization. Fig 7 shows the absorbance spectra obtained for the polyanthracene solutions exposed to LEDs with varying wavelength.

Significant increase in the absorbance in the 262 nm region is evident upon exposure to 381–486 nm LED in Fig 7 compared to a control (not exposed) solution. This increase is dependent on the wavelength of the input light i.e. highest for the 381 nm LED and lowest for the 468 nm LED under similar experiment conditions (60 mins. exposure time, 20 mW irradiation power). An opposite trend can be observed in the peak at approx. 355 nm shown in the inset of Fig 7. The maximum decline in the absorption peak was observed for the sample exposed to 381 nm, indicating maximum product conversion catalyzed or driven by the input light. The presence of an isosbestic point at 345 nm is indicative of a photo-driven equilibrium between the product and the un-dimerized material. The increase in absorbance at 262 nm is indicative of the formation of an ortho-substituted benzene product which has similarity in structure with the expected photo-dimer. No significant change in surface wettability was observed upon exposure to 381 nm compared to UV-C (254 nm) as presented in S4 Fig, suggesting that photo-dimerization dominates over photo-oxidation at higher wavelengths.

**Fig 8:**
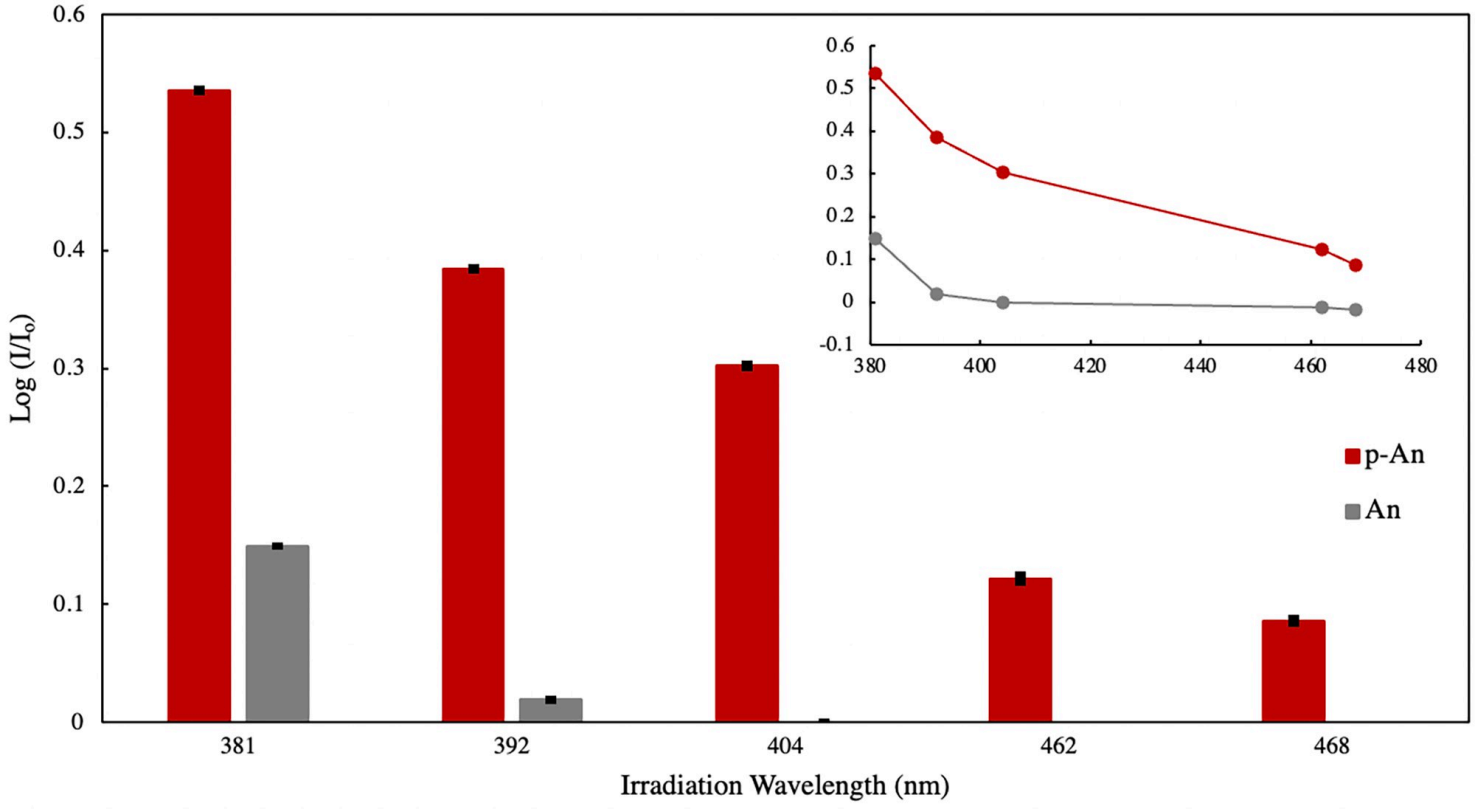
Photo-dimer formation of polyanthracene (red) and anthracene (gray) in ethanol (0.003% w/v) upon exposure to 381–468 nm LEDs (60 mins exposure time, 20 mW irradiation power). Inset shows the reactivity spectra.

The absorbance spectrum of the solution not exposed to any input light (I_o_) served as the benchmark for comparison with the spectrum upon exposure to varying wavelengths in the region 381 to 468 nm. It is expected to have the lowest absorbance at 262 nm, as evident in Fig 7. The absorbance (I) at 262 nm was taken as proportional to the dimer yield which was found to vary with the irradiation wavelength. From the obtained absorbance spectra, log (I/I_o_) was calculated and associated with dimer formation [46]. Employing UV-VIS spectroscopy and measuring change in absorbance before and after irradiation to monitor the progress of the dimerization reaction has also been used in other similar studies [13]. Photo-dimer formation as a function of irradiation wavelength of the input light is shown in Fig 8.

**Fig 9:**
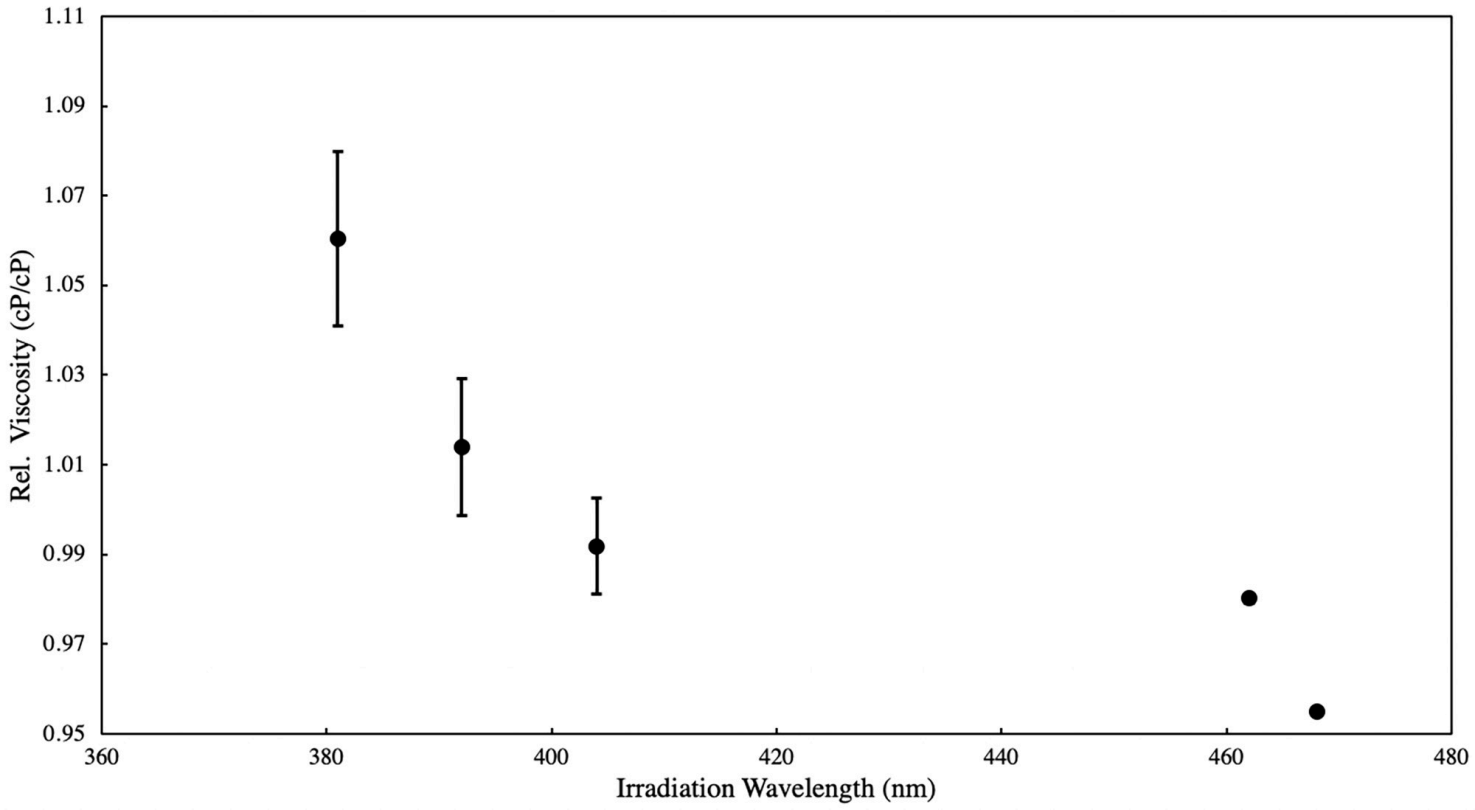
Dynamic viscosity measurements of 0.005% w/v polyanthracene in ethanol solutions exposed to 381–468 nm light as stimulus (60 min irradiation time, 20 mW irradiation power).

A general trend of decrease in photo-dimer product formation was observed from 381 nm to 468 nm. When comparing with data obtained for the monomer, photo-dimer formation upon exposure to 381 nm input light was approx. 4x higher compared to a non-substituted anthracene molecule. Anthracene reports no significant dimerization reaction beyond 404 nm, as opposed to the polymer material. Results show that photo-dimerization of polyanthracene in solution will proceed upon exposure with ultraviolet and visible light LEDs in the region under study, with reduction in efficiency at longer wavelengths. The same trend was observed for other anthracene substituted moieties in literature [47]. Substituted anthracene moieties were observed to have detectable reactivities up to 430 nm after 120 sec of irradiation with a monochromatic pulsed laser, confirming its inherent visible light reactivity. However, the lack of uniform exposure parameters such as type of light source used, its emission spectra (monochromatic, narrowband, or broadband), exposure time and irradiation power renders direct comparison between reactivity plots as inaccurate. To validate the reactivity spectra obtained, dynamic viscosity experiment was performed. Equal amounts of polyanthracene dissolved in ethanol solution were exposed to 381–468 nm light. The input speed of the spindle used is at 200 rpm with viscosity range between 1–150 cP. Measurements were taken at 22°C ambient temperature and plotted relative to the solvent’s viscosity. A similar decreasing trend from 381 nm to 468 nm was observed and shown in Fig 9, consistent with the derived photo-reactivity of the material in the 381–468 nm region. Results suggest that exposure to 381 nm produces more photo-dimer products than those exposed to longer wavelengths, which changes the as-synthesized polymer configuration from a linear and extended conformation to a contracted or coiled structure brought about by the light-catalyzed dimerization reaction among the photo-active groups within the polymer backbone.

Improving the reactivity of light-responsive materials in the visible region is crucial for specific applications such as photo-patterning of cells on functionalized surfaces since UV light may cause irreversible cell damage. Dimerization of functionalized anthracene materials in the visible range (i.e. 410 nm) were similarly reported using catalyst-free triazole-functionalized anthracene materials [23, 24]. This observed red-shift in the reactivity of anthracene towards longer or milder wavelengths was enabled by using electron-rich substituents at either C9 or C10 position of the polyaromatic ring. Though reports also suggest that the kinetics of photo-induced dimerization is compromised in 9-substituted anthracene moieties since the central ring is responsible for its inherent photo-activity [35].

**Fig 10:**
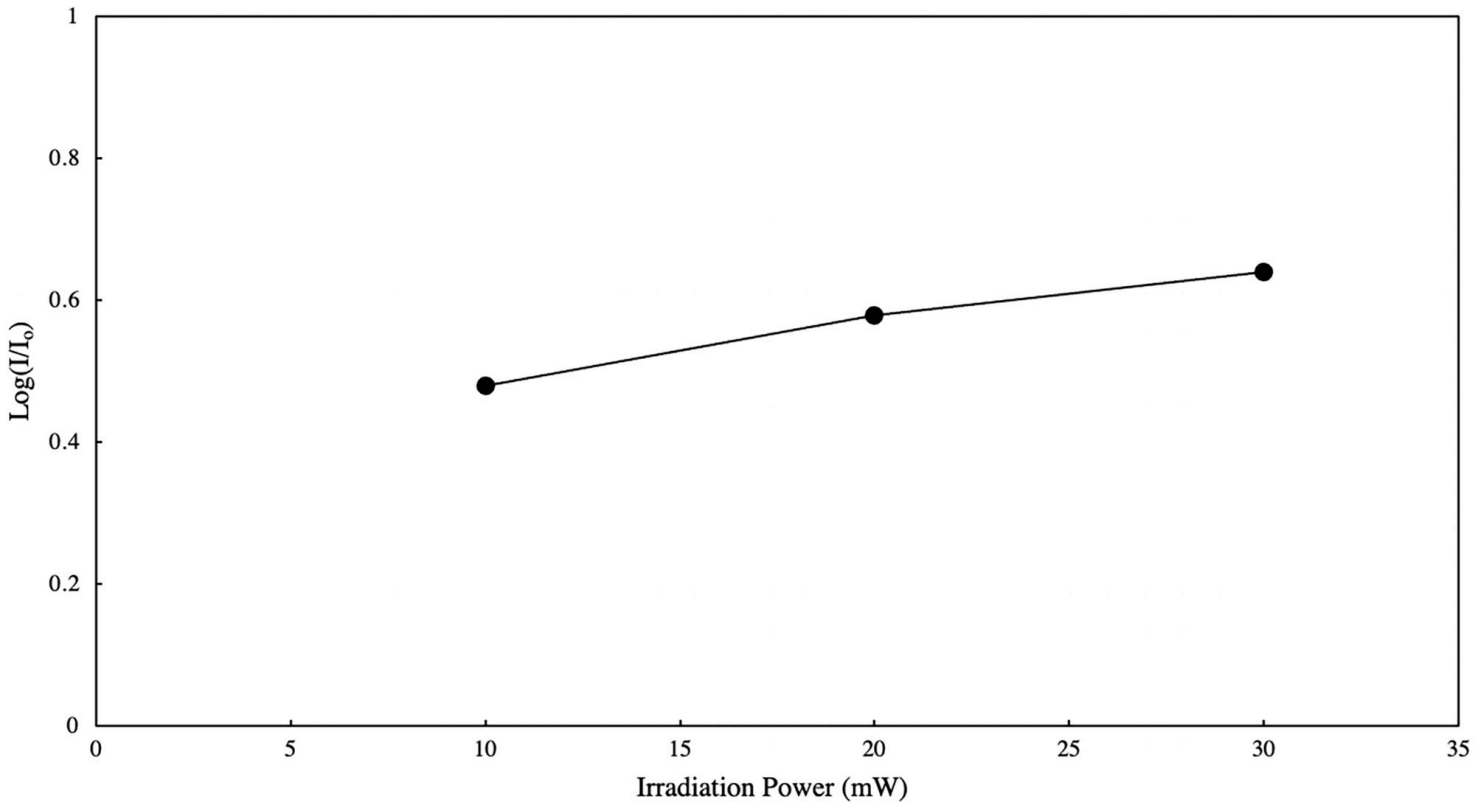
Photo-dimer formation in 0.003% w/v polyanthracene in ethanol solutions exposed to different power output of the 381 nm LED light source (irradiation time 60 mins).

**Fig 11:**
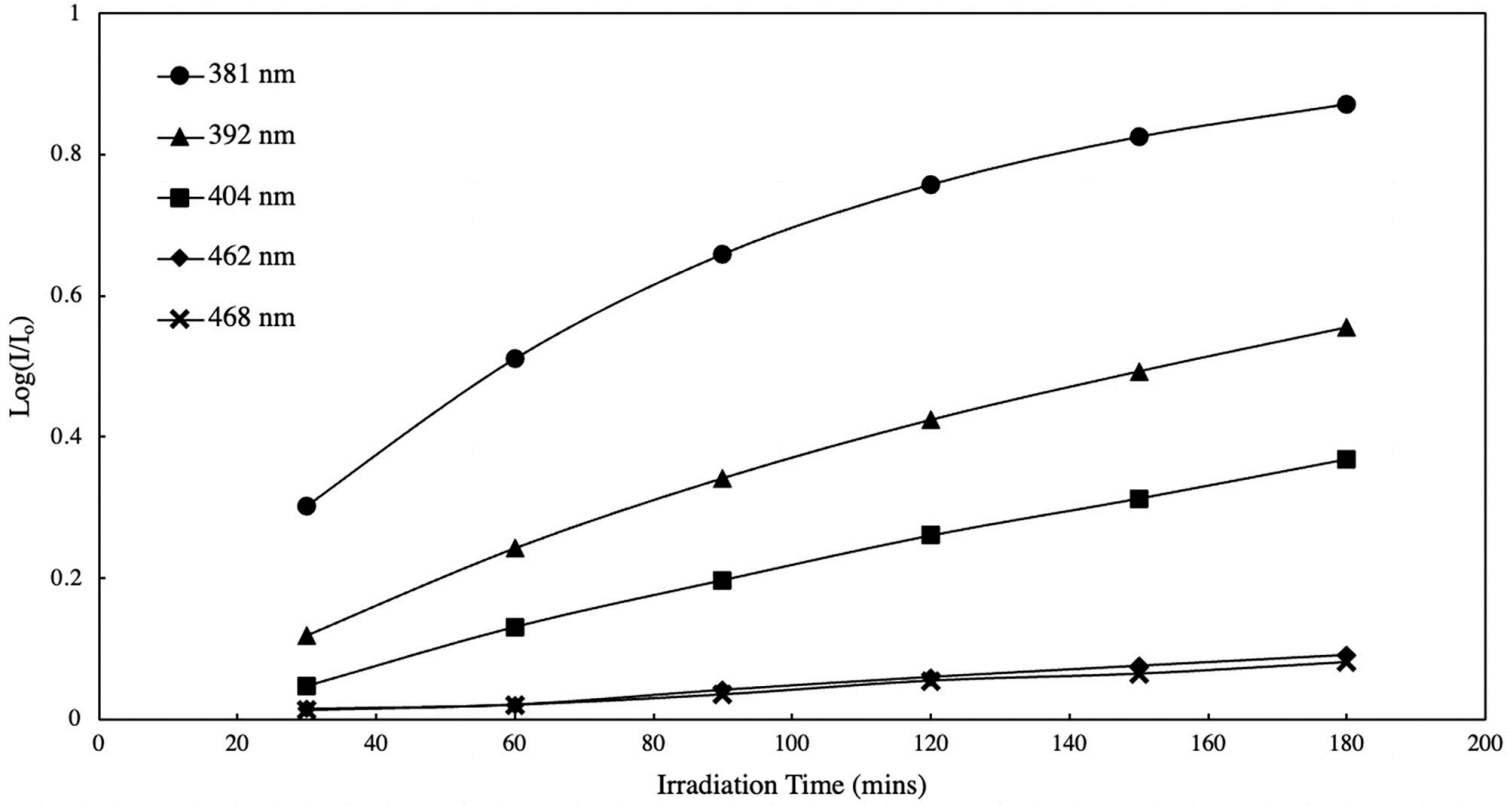
Photo-dimer formation as a function of irradiation time for 0.003% w/v polyanthracene in ethanol solution.

Aside from the wavelength of the light source, other experiment parameters such as irradiation power and irradiation time may be optimized to favor photo-dimer formation and yield. Fig 10 shows the power spectrum obtained for a polyanthracene in ethanol solution exposed to different power output of the light source with constant exposure time (60 mins). The LED light source used was 381 nm since it has the highest reported photo-dimer yield within the region under study. There is an evident increase in photo-dimer formation with increasing power output of the light source. Fig 11 shows the photo-dimer formation as a function of irradiation time (i.e. kinetics plot) obtained for polyanthracene dissolved in ethanol exposed to 381–468 nm LED lights (20 mW). A similar trend was obtained with the variation in power output. These suggest that both power output of the light source and exposure time can be adjusted to promote photo-dimerization of the material polyanthracene in the visible region, where a decrease in reaction efficiency was observed at longer wavelengths in the region under study.

## Conclusion

The current work presented photo-induced reactivity of the material polyanthracene using UV-C and UV-VIS (381–468 nm) light as stimulus. Polyanthracene was synthesized via one-pot chemical oxidation route using FeCl_3_ as oxidizing agent. The material was self-doped with Fe^2+/3+^ and Cl^-^ ions due to the nature of the chemical oxidant used. A decrease in surface hydrophobicity of a polyanthracene-coated PMMA was observed upon UV-C exposure for up to 48 hrs, suggesting increase in oxygen content at the surface. This was validated by elemental analysis of the UV-C exposed polyanthracene using energy dispersive X-ray spectroscopy, providing evidence of material oxidation. Upon exposure to ultraviolet-visible light LEDs, dimerization of polyanthracene in solution occurred and was monitored using UV-VIS spectroscopy. The photo-dimer product formation generally decreased upon light exposure from 381 nm to 468 nm and was found to be higher for polyanthracene compared to the monomer anthracene under similar experiment conditions. Results obtained show that photo-induced dimerization of polyanthracene will proceed upon exposure with visible light LEDs. This observed increase in photo-reactivity in the visible region may be attributed to the presence of electron-rich substituents in the 9 and 10 position of the anthracene moiety of the polymer consistent with those reported in literature. To improve the dimerization efficiency, irradiation power of the light source and irradiation time were increased. While tuning the dimerization wavelength of functionalized or substituted anthracene-based materials towards milder wavelengths may improve bio-conjugation from the perspective of the light source for certain applications, lack of detailed study on the material’s toxicology profile may render the objective futile and thus warrants further scrutiny.

## Supporting information

S1 Fig Exposure set-up using a 2x2 LED array and cross-sectional view of the sample chamber. (TIF)

S2 Fig Emission spectra of the LEDs used in the exposure set-up. (TIF)

S3 Fig Plot of log [n] vs log M_w_ of the polystyrene standards. (TIF)

S4 Fig Change in contact angle over time upon exposure to 254 nm and 381 nm light. (TIF)
