# Supplementary figures and images for: Enhanced photo-reactivity of polyanthracene in the VIS region
Source: PLoS One. 2022 Jul 8;17(7):e0271280. doi: 10.1371/journal.pone.0271280 (PMC9269904; doi:10.1371/journal.pone.0271280)

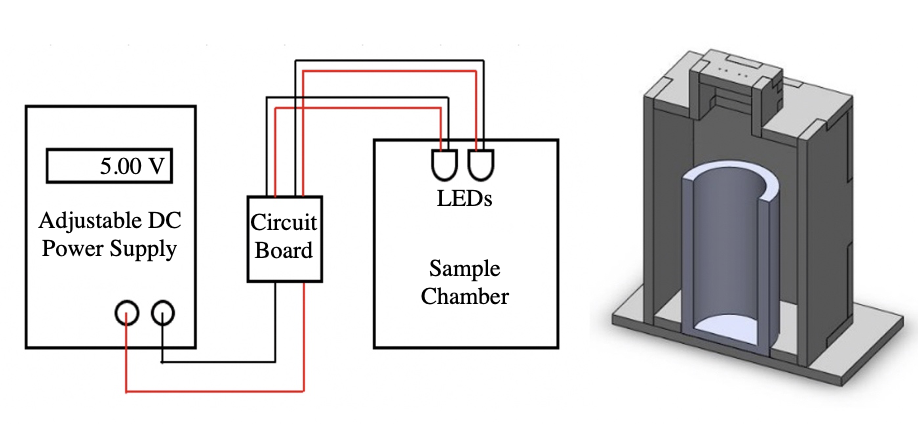

Supplement: S1 Fig — (TIF) [file pone.0271280.s001.tif]

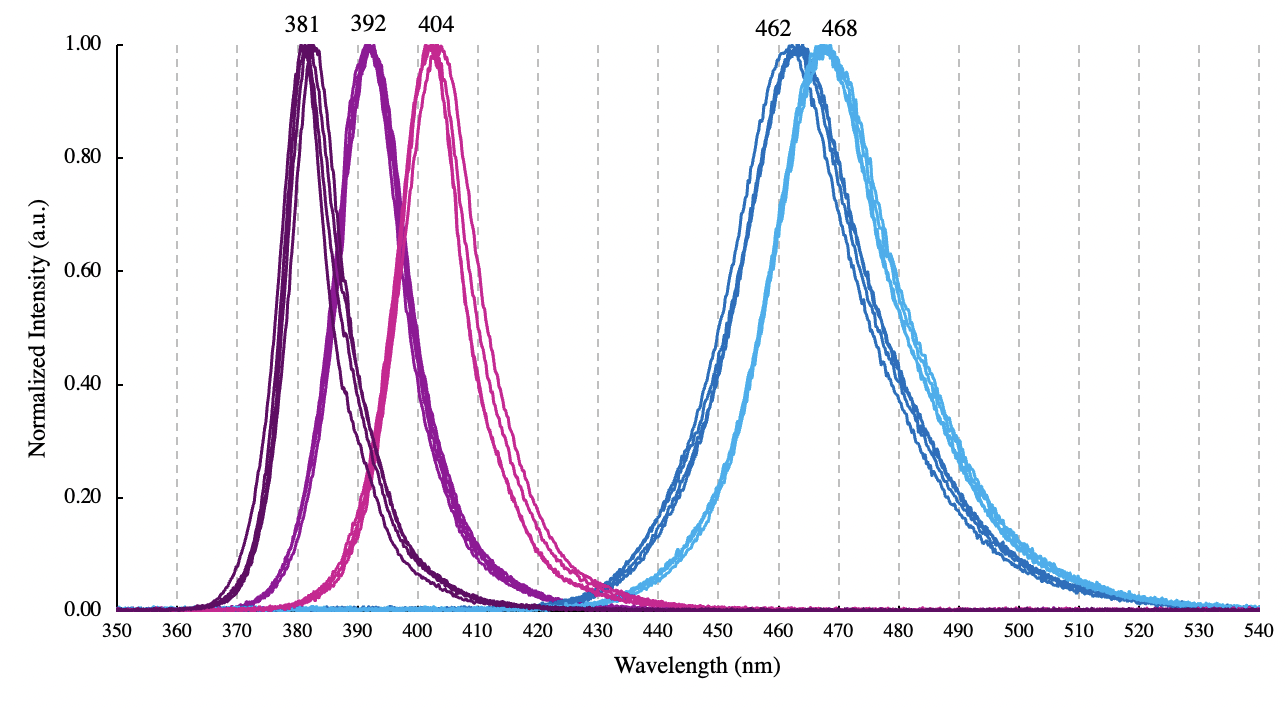

Supplement: S2 Fig — (TIF) [file pone.0271280.s002.tif]

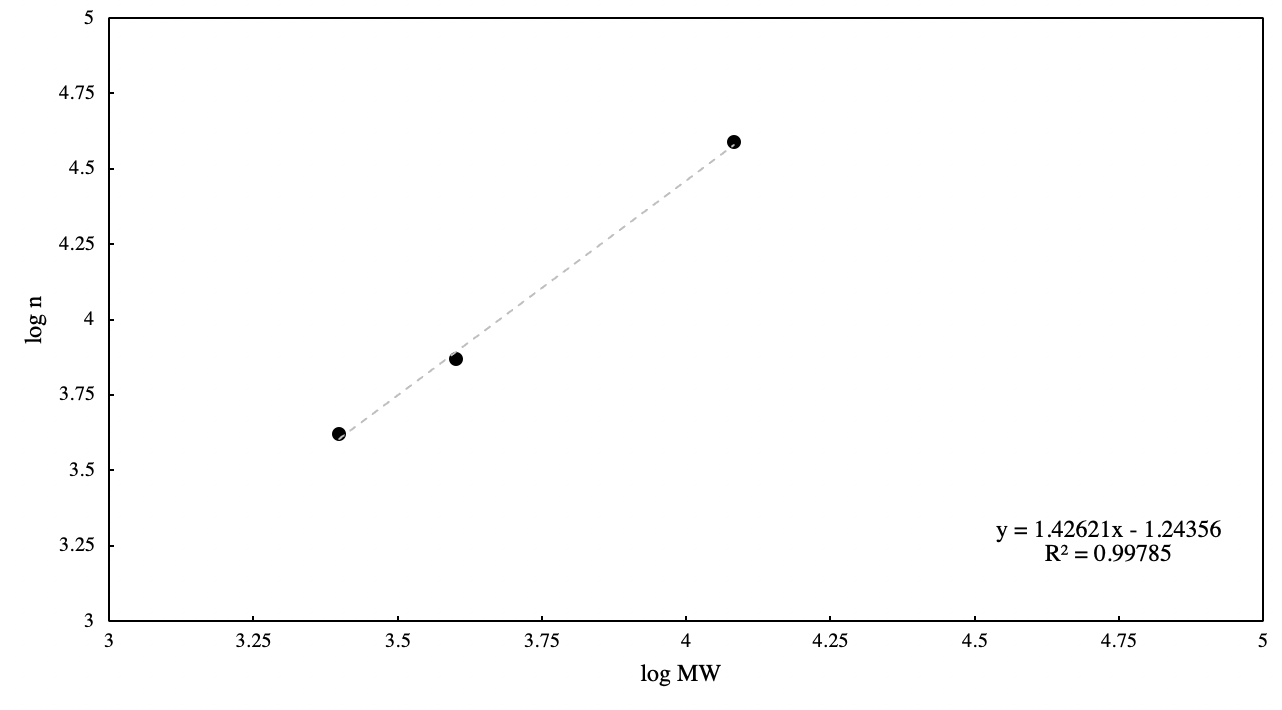

Supplement: S3 Fig — (TIF) [file pone.0271280.s003.tif]

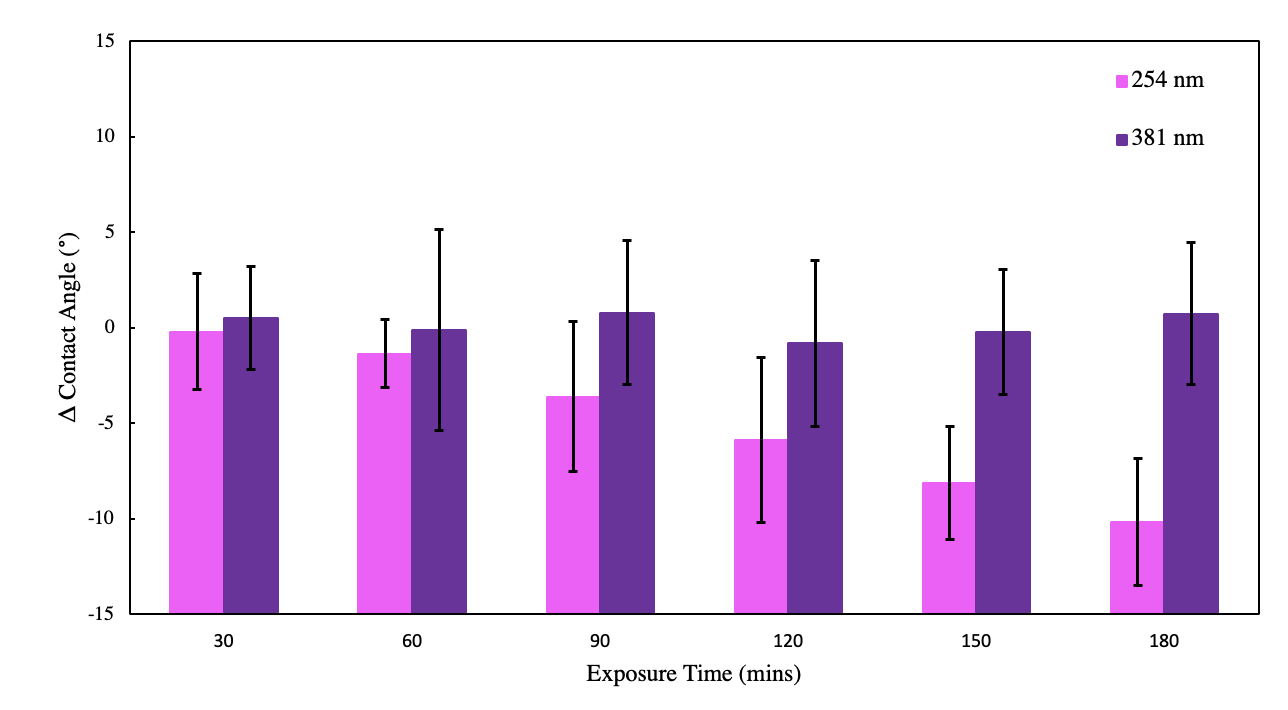

Supplement: S4 Fig — (TIF) [file pone.0271280.s004.tif]
